# Determinants influencing decision-making for operative and perioperative management of grade III and IV hemorrhoidal disease: secondary analysis of a multicenter nationwide prospective cohort study

**DOI:** 10.1007/s00423-026-04030-5

**Published:** 2026-04-15

**Authors:** Metin Kement, Orhan Ali̇moğlu, Hakan Baysal, Salih Tosun, Atif Tekin, Ilker Sucullu, Osman Ci̇vi̇l, Nevi̇n Sakoğlu, Naci̇ye Çi̇ğdem Arslan, Ci̇had Tatar, Rozan Kaya, Ali̇ Emre Nayci, Taygun Gülşen, Serhat Meri̇c, Farid Mohamad Hamad, Ahmed Salhat, Ni̇hat Buğdayci, Sezai̇ Leventoğu, Ramazan Kozan, Özkan Akpinar, Mehmet Karahan, Selçuk Kaya, Hasan Fehmi̇ Küçük, Nai̇l Can Adiguzel, Mustafa Oncel

**Affiliations:** 1 General Surgery Department, Bahcesehir University School of Medicine, VM Medicalpark Pendik Hospital, Kadikoy, Istanbul, Türkiye; 2 General Surgery Department, VM Medicalpark Hospital, Istanbul, Türkiye; 3 General Surgery Department, Medeniyet University, Goztepe Prof. Dr. Suleyman Yalcin City Hospital, Istanbul, Türkiye; 4 General Surgery Department, Medipol University School of Medicine, Medipol Mega Hospital, Istanbul, Türkiye; 5 Sucullu General Surgery Clinic, Istanbul, Türkiye; 6 General Surgery Department, Medipol University School of Medicine, Medipol Bahcelievler Hospital, Istanbul, Türkiye; 7 General Surgery Department, University of Health Sciences, Istanbul Training and Research Hospital, Istanbul, Türkiye; 8 General Surgery Department, Sultanbeyli State Hospital, Istanbul, Türkiye; 9 General Surgery Department, University of Health Sciences, Bagcilar Training and Research Hospital, Istanbul, Türkiye; 10 General Surgery Department, Gazi University School of Medicine, Ankara, Istanbul Türkiye; 11 General Surgery Department, Kartal City Hospital, Health Sciences University, Istanbul, Türkiye

**Keywords:** Hemorrhoidal disease, Hemorrhoidectomy, Hemorrhoid surgery

## Abstract

**Purpose:**

With multiple treatment options available for hemorrhoidal disease (HD), identifying factors that influence surgical and perioperative management decisions is essential, particularly in advanced cases. This study aimed to determine the patient and disease-related determinants affecting the choice of surgical technique and perioperative management in patients with Grade III and IV HD, thereby addressing inter-institutional variations in treatment approaches.

**Methods:**

A secondary analysis was performed on data from a nationwide, multicenter prospective cohort study. The study included 315 patients diagnosed with Grade III (72%) and Grade IV (28%) HD, with a mean age of 43.7 ± 11.4 years and a male predominance (76.7%). Preoperative data, including patient demographics, comorbidities (ASA scores), symptom severity, and clinical findings, were collected at participating governmental and private hospitals. Surgical techniques were classified as excisional or non-excisional, while anesthesia type, use of perianal or pudendal analgesia, and hospitalization duration were determined by the surgical teams. Hospital type was also recorded. Multivariable analyses were conducted to identify factors influencing the choice of surgical techniques, anesthesia, analgesia application, and the decision for outpatient procedures.

**Results:**

Multivariate analysis revealed that the presence of thrombosis significantly influenced the choice of surgical technique (OR: 7.2, CI: 2.8–12.7, *p* = 0.001), while hospital category also played an important role (OR: 5.1, CI: 2.7–9.7, *p* = 0.001). For anesthesia type, factors such as disease grade (OR: 3.3, CI: 1.6–6.7, *p* = 0.001), hospital category (OR: 9, CI: 4.1–19.9, *p* < 0.001), and surgical technique (OR: 6.8, CI: 3–15.3, *p* < 0.001) were significant determinants. The decision to use perianal or pudendal analgesia was influenced by hospital category (OR: 27.1, CI: 11.7–62.6, *p* < 0.001) and the presence of incontinence (OR: 0.2, CI: 0.04–0.93, *p* = 0.04). Outpatient management was associated with disease grade (OR: 2.3, CI: 1.1–4.8, *p* = 0.023), hospital category (OR: 2, CI: 1.2–3.2, *p* = 0.011), higher comorbidity (ASA ≥ 3, OR: 3.3, CI: 1.8–3.2, *p* = 0.007), and the selected surgical technique (OR: 3.1, CI: 1.6–5.8, *p* = 0.001).

**Conclusion:**

Significant inter-institutional variations exist in the management of advanced HD. Among various factors, the presence of thrombosis emerges as the predominant determinant in surgical decision-making, providing valuable insights for standardizing treatment protocols and reducing practice variability.

**Supplementary Information:**

The online version contains supplementary material available at 10.1007/s00423-026-04030-5.

## Introduction

Hemorrhoidal disease (HD) is a common anorectal condition with a significant impact on patient quality of life. Despite its prevalence, the management of HD exhibits considerable variability in clinical practice, influenced by diverse therapeutic approaches, regional healthcare systems, and evolving treatment technologies [1–5]. The Goligher classification, a four-grade system for categorizing the severity of hemorrhoidal prolapse, is widely recognized as a crucial determinant in treatment decision-making, particularly in the selection of surgical technique [6].

Current clinical guidelines recommend a range of surgical approaches for the management of Grade III and IV hemorrhoids, including excisional hemorrhoidectomy, stapled hemorrhoidopexy, doppler-guided hemorrhoidal artery ligation, and ablative techniques [7]. The management of symptomatic hemorrhoids necessitates an individualized approach. Our findings support the established role of conventional hemorrhoidectomy, considered the gold standard for more symptomatic grade 3 and 4 hemorrhoids. Patients with higher *Hemorrhoidal Disease Symptom Score (HDSS) and Short Health Scale-Hemorrhoidal Disease (SHS-HD)* scores, indicative of more severe disease, were more likely to undergo excisional hemorrhoidectomy in our cohort, aligning with the recognized clinical practice for managing patients with significant symptoms [8–10]. Each surgical modality presents distinct advantages and limitations with respect to postoperative pain, quality of life, recovery time, and cost [8–10]. Despite advancements in surgical techniques, considerable variability exists in the perioperative management of HD across clinical settings. Prospective randomized trials have consistently demonstrated the superiority of several anesthetic approaches, including general anesthesia with local infiltration, over spinal anesthesia alone. Other applications include the use of adjunctive analgesia such as pudendal nerve block or perianal injections of local anesthetics for improved postoperative pain control [11–13]. Advancements in surgical techniques, such as minimally invasive procedures and enhanced postoperative pain management, have made outpatient hemorrhoidectomy a feasible and increasingly common option [14]. Despite the evidence supporting these practices, there is a significant gap in our understanding of their real-world application. This variability in clinical practice may be influenced by a multitude of factors, including institutional policies, surgeon expertise, patient preferences, and the characteristics of the healthcare system. However, the specific factors that drive clinician decision-making in the selection of these different management approaches remain largely unexplored. The significant variability in the management of HD across different settings highlights the crucial importance of conducting multicenter analyses to identify patterns, discrepancies, and ultimately establish best practices in the field. Utilizing data from a multicenter nationwide prospective cohort study, this study aims to investigate the factors that influenced surgical decision-making in the treatment of HD, including the choice of surgical techniques and the implementation of various perioperative applications.

## Methods

A prospective cohort study was conducted between July 2022 and July 2024 at 20 tertiary care or university hospitals across diverse regions at Türkiye (registered on ClinicalTrials.gov with the identifier number of NCT05429060). Istanbul Medipol University Research Committee granted the study and Institutional Ethics Committee approved the protocol (Protocol number: E-10840098-772.02-3634) in compliance with the Declaration of Helsinki. This study presents a secondary analysis of abstracted information to identify the factors influencing surgeons’ preferences for surgical approaches. To ensure data homogeneity, we included only patients with *Grade III or IV* disease and centers that contributed at least 10 cases to the study.

Patients aged 18 years and older who underwent elective surgery for symptomatic hemorrhoidal disease were included. Urgent or emergent procedures were excluded from the study. Exclusion criteria included patients with concurrent proctological conditions such as fistulas, abscesses, and fissures; a history of prior hemorrhoid surgery; inflammatory bowel disease; pelvic or perineal radiation; previous perianal trauma or obstetric injury; and prior rectal surgeries for benign or malignant conditions. To ensure standardized treatment, all patients were required to undergo at least four weeks of conservative management prior to surgical intervention. This prerequisite was intended to ensure that patients had failed conservative therapy before being offered surgery, consistent with guideline-based care, rather than to standardize the content of the conservative treatment itself. Sigmoidoscopy was required for patients under 50, while colonoscopy was mandatory for those over 50 to rule out other potential pathologies. All perioperative process including surgical technique, anesthesia type, application of pudendal or perianal analgesic application and outpatient hospitalization was decided by the surgical teams or surgeons.

Data collection included patient demographics, body mass index, anticoagulant use, comorbidities (American Society of Anesthesiology (ASA) Score), smoking, and delivery history. Surgical teams also recorded patient complaints (pain, itching, bleeding, prolapse, soiling, tenesmus, constipation, and incontinence), physical examination findings (prolapse, skin tags, thrombus, bleeding, soiling/discharge). The presence of thrombosis was determined by clinical examination, defined as the identification of a firm, tender, bluish subcutaneous perianal mass consistent with thrombosed hemorrhoidal tissue., and disease severity using the Goligher classification [6]. Two different questionnaires were used for reporting patient reported outcome measures (PROMs). The severity of hemorrhoidal symptoms was assessed using the Hemorrhoidal Disease Symptom Score (HDSS), a 5-item scale with a possible range from 0 to 20 [15]. Health-related quality of life associated with hemorrhoids was evaluated using the Short Health Scale-Hemorrhoidal Disease (SHS-HD), a 4-item scale with a possible range from 0 to 21 [16]. The experience level of the operating surgeon, whether attending staff or supervised resident, was recorded.

According to study protocol, perioperative management decisions, including surgical technique, anesthesia type, application of pudendal analgesia or perianal analgesics, and time for discharge were left to the discretion of the surgical teams. Surgical techniques were categorized as excisional (Milligan-Morgan and Ferguson hemorrhoidectomy) or non-excisional (stapler hemorrhoidopexy, Doppler-guided hemorrhoidal artery ligation [HAL] with or without mucopexy, and laser [HeLP]). Other perioperative measures including anesthesia type (general, spinal/regional), the application of perianal anesthetics or pudendal block, and outpatient status following surgery were documented. The participating hospital where the surgery was performed, as well as hospital category (governmental or private), was recorded.

### Statistics

All analyses were performed using SPSS version 26. Continuous variables were presented as mean ± standard deviation, and categorical variables as frequencies and percentages.Group comparisons were conducted using one-way ANOVA for normally distributed data and the Kruskal-Wallis test for non-normally distributed data. Categorical variables were analyzed using chi-square or Fisher’s exact tests, with adjusted residuals explored for significant associations.Binary logistic regression was used to identify factors influencing surgical and perioperative decisions. Variables with a univariate *p* < 0.2 were included in multivariate models. A *p* < 0.05 threshold was considered statistically significant. Multicollinearity was evaluated using the variance inflation factor (VIF), with a threshold of VIF > 5 indicating potential multicollinearity. No significant multicollinearity was detected among the variables included in the final models. Given the hierarchical structure of the data with patients nested within institutions, a sensitivity analysis using generalized estimating equations (GEE) was also performed to account for potential clustering effects at the institutional level.

## Results

Out of the 315 patients operated on across 20 participating centers, only 279 patients (88.6%) from 9 institutions were eligible for inclusion in the study. The mean age of patients was 43.7 ± 11.4 years with male majority (*n* = 214, 76.7%). All included centers were tertiary-level governmental (*n* = 6) or private (*n* = 3) institutions affiliated to universities or education and research hospitals, providing a consistent standard of care and resource availability. Grade III hemorrhoids were observed in 201 (72%) patients, and Grade IV in 78 (28%). Surgical procedures were performed in 170 (60.9%) patients at governmental hospitals and 109 (39.1%) at private institutions. Demographic and perioperative data for the cohort, encompassing both grade III and grade IV disease, are summarized in Table 1.

**Table 1:** Patient characteristics and perioperative data for the cohort

Studied Parameters	Grade III (*n* = 201)	Grade IV (*n* = 78)	Study Cohort (*n* = 279)
Patient Characteristics			
Gender (male, %)	152(75.6)	62 (79.4)	214 (76.7)
Age (mean years ±SD)	44.3±11.4	42±11.4	43.6±11.4
BMI (mean kg/m2 ±SD)	26.1±3.8	25.2±3.4	25.9±3.6
Comorbidity (ASA ≥ 3) (n, %)	23 (11.4%)	10 (12.8%)	33 (11.8)
Anti-coagulant (n, %)			11 (3.9)
Smoking (n, %)	61 (30.3%)	26 (33.3%)	87 (31.2)
Delivery History *n* = 65 F (n, %)	25(51 of F)	10(62.5 of F)	35 (52.8)
Patient Complaints			
Pain (n, %)	148 (73.6%)	58 (74.4%)	206 (73.8)
Itching (n, %)	94 (46.8%)	33 (42.3%)	127 (45.5)
Bleeding (n, %)	137 (68.2%)	57 (73.1%)	194 (69.5)
Prolapse (n, %)	82 (40.8%)	56 (71.8%)	138 (49.5)
Soiling (n, %)	73 (36.3%)	36 (46.2%)	109 (39.1)
Tenesmus (n, %)	55 (27.4%)	24 (30.8%)	79 (28.3)
Constipation (n, %)	99 (49.5%)	38 (48.7%)	137 (49.1)
Incontinence (n, %)	13 (6.5%)	10 (12.8%)	23 (8.2)
Physical Examination Findings			
Prolapse (n, %)	68 (33.8%)	53 (67.9%)	121 (43.4)
Skin Tag (n, %)	61 (30.3%)	26 (33.3%)	87 831.2)
Thrombus (n, %)	34 (16.9%)	19 (24.4%)	53 (19)
Bleeding (n, %)	84 (41.8%)	41 (52.6%)	125 (44.8)
Soiling/Discharge (n, %)	62 (31.0%)	29 (37.2%)	91 (32.6)
PROMs			
Preop. SHS-HD(mean±SD)	16.5±5.3	18.1±5.0	16.9±5.2
Preop. HDSS (mean±SD)	10.5±4.7	10.7±4.1	10.6±4.5
Hosiptals			
Governmental Institutions			
#1	26 (12.9%)	6 (7.7%)	32 (11.4)
#3	8 (4.0%)	2 (2.6%)	10 (3.6)
#4	65 (32.3%)	8 (10.3%)	73 (26.1)
#5	19 (9.5%)	10 (12.8%)	29 (10.4)
#6	10 (5.0%)	6 (7.7%)	16 (5.7)
#9	0 (0.0%)	10 (12.8%)	10 (3.6)
Private Institutions			
#2	14 (7.0%)	9 (11.5%)	23 (8.2)
#7	53 (26.4%)	20 (25.6%)	73 (26.1)
#8	6 (3.0%)	7 (9.0%)	13 (4.7)
Hospital Category			
Government	128 (63.7%)	42 (53.8%)	170 (60.9)
Private	73 (36.3%)	36 (46.2%)	109 (39.1)
Surgical Technique			
Excisional	129 (64.2%)	57 (73.1%)	186 (66.7)
Non-Excisional	72 (35.8%)	21 (26.9%)	93 (33.3)
Anesthesia Type			
General	132 (65.7%)	38 (48.7%)	170 (60.9)
Spinal/Regional	69 (34.3%)	40 (51.3%)	109 (39.1)
Application of Perianal Analgesics or Pudendal Anesthesics			
Yes	56 (27.9%)	30 (38.5%)	86 (30.8)
No	145 (72.1%)	48 (61.5%)	193 (69.2)
Outpatient Procedures			
Yes	67 (33.3)	13 (16.7)	80 (28.7)
No	134 (66.7)	65 (83.3)	199 (71.3)

(*PROM* Patient reported outcome measure, *HDSS* Hemorrhoidal Disease Severity Score, *SHS-HD* Short Health Scale-Hemorrhoidal Disease)

### Decision-making for operative technique

**Fig. 1:**
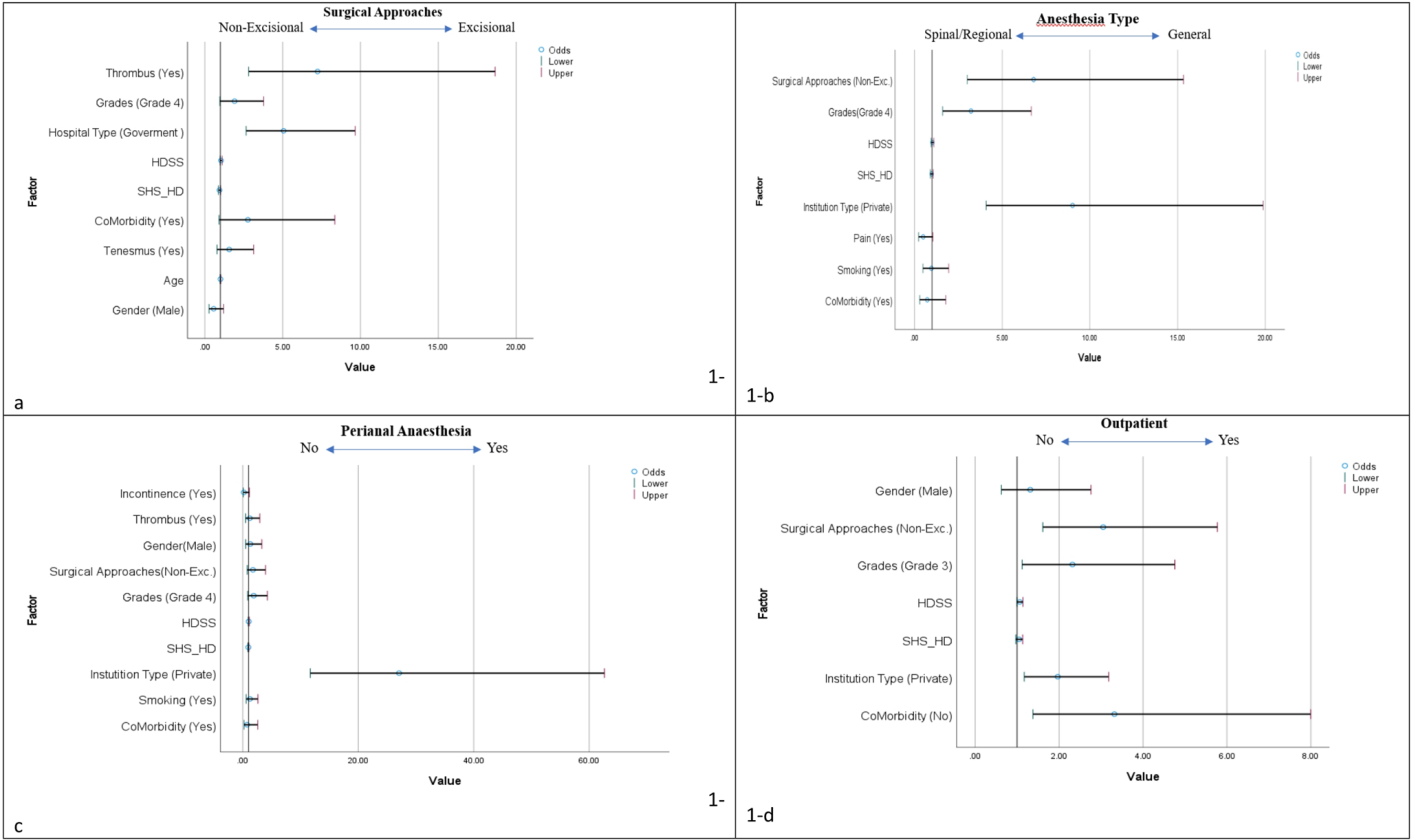
Forrest Plot of multivariate logistic regression analysis of variables influencing decision-making for operative and perioperative outcome measures (1-a: Surgical Technique, 1-b: Anesthesia Type, 1-c: Application of Perianal Analgesics Injection or Pudental Anesthesia, and 1-d: Outpatient Hospitalization)

Excisional techniques (Milligan-Morgan [*n* = 142, 76.3%], Ferguson [*n* = 44, 23.6%]) were preferred in 186 (66.7%) patients, while non-excisional techniques (stapler hemorrhoidopexy: *n* = 3, 3.2%; HAL with/without mucopexy: *n* = 17, 18.3%; HeLP: *n* = 73, 78.5%) were favored in 93 (33.3%). Further analysis showed a trend towards a higher utilization of non-excisional techniques in males (*p* = 0.045) and a significant association between co-morbidities and the use of excisional techniques (*p* = 0.049) (Table 1). Patients who reported tenesmus (*p* = 0.019) or had a thrombosed hemorrhoid (*p* = 0.013) on physical examination were significantly more likely to be treated with excisional techniques. While *Goligher* score did not significantly influence surgical technique (*p* = 0.157), patients with higher HDSS (*p* = 0.01) and SHS-HD scores (*p* = 0.049) were more likely to undergo excisional procedures (Table 2). The choice of surgical technique varied significantly across the 9 different participating institutions (*p* < 0.001), and institutional type, whether governmental or private, was a significant determinant of the surgical approach employed (*p* < 0.001, Table 2). There were significant inter-hospital variations for both governmental and private institutions (*p* < 0.001 for both). Multivariate analysis demonstrated that the presence of thrombosed hemorrhoids and the hospital category significantly influenced the choice of surgical technique (both *p* = 0.001) (Table 3; Fig. 1-a).

**Table 2:** Influence of studied parameters on clinical decision-making for operative and periopeative outcome measures

Variables	Surgical Approaches			Anaesthesia Type			Perianal-Pudendal Anaesthesia			Outpatient Hospitalization		
	Excisional (*n*=186)	Non-Excisional (*n*=93)	*p*	General (*n*=170)	Spinal/Regional (*n*=109)	*P*	Yes *n*=86	No *n*=193	*P*	Yes (*n*=80)	No (*n*=199)	*P*
Patient Characteristics												
Gender (male, %)	136 (73)	78 (83.9)	0.045*	134 (78.8)	80 (73.4)	0.295	72 (83.7)	141 (73.8)	0.071*	66 (82.5)	148 (74.4)	0.146*
Age (mean years ±SD)	44.3±11.9	42.7±10	0.087*	43.2±10.4	44.4±12.8	0.46	46.1±11.8	38±7.9	<0.001*	44.9±11.4	43.1±12	0.519
BMI (mean kg/m2 ±SD)	26.2±3.9	25.2±3.1	0.64	25.9±4.1	25.7±3.2	0.77	26.3±4.1	25.7±3.2	0.54	26.6±4.1	25.5±3.2	0.35
Comorbidity (ASA ≥ 3) (n, %)	27 (14.5)	6 (6.5)	0.049*	15 (8.8)	18 (16.5)	0.052*	7 (8.1)	26 (13.6)	0.193*	14 (17.5)	19 (9.5)	0.063*
Anti-coagulant (n, %)	8 (4.3)	3 (3.2)	0.66	7 (4.1)	4 (3.7)	0.85	9 (4.7)	2 (2.3)	0.347	4 (5)	7 (3.5)	0.565
Smoking (n, %)	55 (29.5)	32 (34.4)	0.41	61 (35.9)	26 (23.9)	0.034*	47 (37.9)	40 (20.8)	0.027*	28 (35)	59 (29.6)	0.383
Delivery History n=65 F (n, %)	26 (52)	9 (60)	0.53	21 (58.3)	14 (48.3)	0.41	8 (57.1)	27 (51.9)	0.728	11 (13.8)	24 (12.1)	0.7
Patient Complaints												
Pain (n, %)	138 (74.2)	68 (73.1)	0.847	117 (68.8)	89 (81.7)	0.017*	64 (74.4)	142 (73.6)	0.882	56 (70)	150 (75.4)	0.355
Itching (n, %)	79 (45.5)	48 (51.6)	0.248	77 (45.3)	50 (45.9)	0.925	45 (52.3)	82 (42.5)	0.228	40 (50)	87 (43.7)	0.323
Bleeding (n, %)	127 (68.3)	67 (72)	0.52	112 (65.9)	82 (75.2)	0.098	139 (72)	55 (64)	0.276	55 (68.8)	139 (69.8)	0.857
Prolapse (n, %)	92 (49.5)	46 (49.5)	1.0	83 (48.8)	55 (50.5)	0.789	103 (53.4)	35 (40.7)	0.051*	38 (47.5)	100 (50)	0.82
Soiling (n, %)	70 (37.6)	39 (41.9)	0.488	61 (35.9)	48 (44)	0.273	76 (39.4)	33 (38.4)	0.87	32 (40)	77 (38.7)	0.84
Tenesmus (n, %)	61 (32.8)	18 (19.4)	0.019*	47 (27.6)	32 (29.4)	0.756	26 (30.2)	51 (26.4)	0.511	21 (26.3)	58 (29.1)	0.627
Constipation (n, %)	91 (48.9)	46 (50)	0.866	81 (47.6)	56 (51.4)	0.903	44 (51.2)	93 (48.4)	0.674	36 (45)	101 (50.7)	0.384
Incontinence (n, %)	13 (7)	10 (10.8)	0.281	13 (7.6)	10 (9.2)	0.651	3 (3.5)	20 (10.4)	0.054*	5 (6.3)	18 (9)	0.443
Physical Examination Findings												
Prolapse (n, %)	87 (46.8)	34 (36.6)	0.105*	73 (42.9)	48 (44)	0.378	42 (48.8)	79 (40.9)	0.218	35 (43.8)	86 (43.2)	0.935
Skin Tag (n, %)	64 (34.4)	23 (24.7)	0.10*	50 (29.4)	37 (33.9)	0.425	27 (31.3)	60 (36.2)	0.959	22 (27.5)	65 (32.5)	0.4
Thrombus (n, %)	43 (23.1)	10 (10.8)	0.013*	33 (19.4)	20 (18.3)	0.825	24 (27.9)	29 (15)	0.011*	16 (20)	37 (18.6)	0.786
Bleeding (n, %)	87 (46.8)	38 (40.9)	0.349	79 (46.5)	46 (42.2)	0.425	38 (44.2)	87 (45)	0.89	35 (43.8)	90 (45.2)	0.823
Soiling/Discharge (n, %)	65 (35.1)	26 (28.0)	0.229	53 (31.2)	38 (34.9)	0.521	27 (31.4)	64 (33.3)	0.75	28 (35)	63 (31.8)	0.609
Physical Examination-Disease Grade (Golligher Classification)												
III (n, %)	129 (69.4)	72 (77.4)	0.157*	132 (77.6)	69 (63.3)	0.009*	56 (65.1)	145 (75.1)	0.085*	67 (83.8)	134 (67.3)	0.006*
IV (n, %)	57 (30.6)	21 (22.6)		38 (22.4)	40 (36.7)		30 (34.9)	48 (24.9)		13 (16.3)	65 (32.7)	
PROMs												
Preop. HDSS (mean±SD)	11.1±4.7	9.6±4	0.01*	10 ±4.7	11.5±4.1	0.013*	9.5±4.3	11.1±4.6	0.09*	9.1±4.3	11.2±4.5	<0.001*
Preop. SHS-HD (mean±SD)	17.3±4.9	16.4±5.7	0.049*	16.5±5.6	17.8±4.5	0.036*	15.9±4.9	17.5±5.3	0.02*	15.1±5	17.1±5.1	<0.001*
Hospitals (both Governmental and Private)			<0.001			<0.001			<0.001			<0.001
Governmental Hospitals			<0.001			<0.001			<0.001			<0.001
#1 (n=32)	31 (96.9)	1 (3.1)		5 (15.6)	27 (84.4)		0	32 (100)		9 (28.1)	23 (71.9)	
#3 (n=10)	10 (100)	0		1 (10)	9 (90)		0	10		1 (10)	9 (90)	
#4 (n=73)	44 (60.3)	29 (39.7)		65 (89)	8 (11)		0	73 (100)		16 (21.9)	57 (78.1)	
#5 (n=29)	24 (82.8)	5 (17.2)		1 (3.4)	28 (96.6)		3 (10.3)	26 (89.7)		3 (10.3)	26 (89.7)	
#6 (n=16)	16 (100)	0 (0)		1 (6.3)	15 (93.7)		0	16 (100)		10 (62.5)	6 (37.5)	
#9 (n=10)	10 (100)	0 (0)		1 (10)	9 (90)		6 (60)	4 (40)		0	10 (100)	
Private Hospitals			<0.001			<0.001			<0.001			<0.001
#2 (n=23)	11 (47.8)	12 (52.2)		22 (95.7)	1 (4.3)		13 (56.5)	10 (43.5)		1 (4.3)	22 (95.7)	
#7 (n=73)	29 (39.7)	44 (60.3)		73 (100)	0		57 (78.1)	16 (21.9)		37 (50.7)	36 (49.3)	
#8 (n=13)	11 (84.6)	2 (15.4)		1 (7.7)	12 (92.3)		7 (53.9)	6 (46.1)		4 (30.8)	9 (69.2)	
Hospital Category												
Government (n=170)	135 (72.6)	35 (37.6)	<0.001*	74 (43.5)	96 (56.5)	<0.001*	9 (5.4)	161 (94.6)	<0.001*	38 (22.4)	132 (77.6)	0.003*
Private (n=109)	51 (27.4)	58 (62.4)		96 (88.1)	13 (11.9)		75 (70.7)	34 (29.3)		42 (38.5)	67 (61.5)	
Surgical Technique												
Excisional	NA		NA	87 (51.2)	10 (9.2)	<0.001*	40 (46.5)	47 (24.4)		3 (45)	49 (24.6)	<0.001*

(*PROM* Patient reported outcome measure, *HDSS* Hemorrhoidal Disease Severity Score, *SHS-HD* Short Health Scale-Hemorrhoidal Disease, *NA* Not applicable. Statistically significant p values were mentioned in bold. (*): p<0.2, and selected for multivariate logistic regression)

**Table 3:** Multivariate logistic regression analysis of variables influencing the decision-making for surgical technique

Variables	B	*p*-value (Sig.)	Odds Ratio (Exp(B))	95% Confidence Interval
Gender (Male)	-0.562	0.140	0.570	0.270–1.203
Age	0.008	0.572	1.008	0.981–1.036
Thrombus (Yes)	1.981	**0.001**	7.247	2.815–12.653
Tenesmus (Yes)	0.451	0.201	1.570	0.786–3.135
Comorbidity (ASA ≥ 3)	1.018	0.071	2.768	0.917–8.354
Preoperative HDSS	-0.055	0.163	0.946	0.876–1.023
Preoperative SHS-HD	0.042	0.211	1.043	0.976–1.114
Hospital Category (Governmental)	1.622	**0.001**	5.065	2.656–9.656
Disease Grade (Grade 4)	0.649	0.061	1.914	0.970–3.778

(Statistically significant p values were mentioned in bold.)

### Decision-making for other perioperative measures

Current study also investigated the factors influencing the selection of anesthesia type, the application of perianal anesthetics or pudendal block, and the decision for outpatient surgery. Patient characteristics influenced perioperative management decisions. Smoking status significantly influenced the choice of anesthesia type (*p* = 0.034), while both smoking status and patient age were associated with the decision to administer perianal analgesia or pudendal block (*p* = 0.27 and *p* < 0.001, respectively). Patients experiencing pain were significantly more likely to undergo surgery under spinal/regional anesthesia (*p* = 0.017), while those with thrombosed hemorrhoids were more frequently administered perianal or pudendal analgesia (*p* = 0.011). Patients with Grade IV hemorrhoids were more likely to require general anesthesia and less likely to be discharged as outpatients compared to those with Grade III disease. Preoperative HDSS and SHS-HD scores, institutional factors (hospital and hospital category), and the type of surgical procedure (excisional vs. non-excisional) were significant predictors of perioperative management decisions. Significant inter-institutional variations were observed across all outcome measures in both governmental and private institutions (*p* < 0.001 for all) (Table 2).

Multivariate analysis revealed that hospital category (*p* < 0.001), disease severity (*p* = 0.001), and the surgical technique employed (*p* < 0.001) were significant predictors of anesthesia type. Hospital category (*p* < 0.001) and the presence of fecal incontinence (*p* = 0.04) were significantly associated with the use of perianal or pudendal analgesia. Finally, comorbidities (*p* = 0.007), hospital category (*p* = 0.011), disease severity (*p* = 0.023), and the surgical technique utilized (*p* = 0.001) significantly influenced the decision for outpatient hospitalization following hemorrhoidectomy (Table 4, and Fig. 1-b, 1-c and 1-d).

**Table 4:** Multivariate logistic regression analysis of variables influencing decision-making for perioperative outcome measures

Variables	B	*p*-value (Sig.)	Odds Ratio (Exp(B))	95% Confidence Interval
Type of Anaesthesia				
Comorbidity (ASA ≥ 3)	-0.317	0.488	0.728	0.297–1.786
Smoking (Yes)	-0.035	0.923	0.966	0.478–1.953
Pain (Yes)	-0.704	0.067	0.495	0.233–1.049
Institution Category (Private)	2.199	**0.000**	9.012	4.087–19.870
Preoperative HDSS	-0.019	0.642	0.981	0.904–1.064
Preoperative SHS_HD	0.021	0.553	1.022	0.952–1.097
Disease Grades (Grade 3)	-1.180	**0.001**	3.33	1.577–6.666
Surgical Technique (Non-Excisional)	1.916	**0.000**	6.792	3.008–15.336
Application of Perianal Analgesics Injection or Pudendal Anesthesia				
Comorbidity (ASA ≥ 3)	-0.294	0.643	0.745	0.215–2.586
Smoking (Yes)	0.218	0.569	1.243	0.587–2.631
Institution Category (Private)	3.299	**0.000**	27.078	11.704–62.647
Preoperative HDSS	-0.030	0.523	0.970	0.884–1.065
Preoperative SHS-HD	0.031	0.449	1.032	0.951–1.119
Disease Grade (Grade 4)	0.643	0.118	1.902	0.849–4.261
Surgical Technique (Non-excisional)	0.566	0.170	1.762	0.784–3.956
Gender (Male)	0.264	0.577	1.303	0.514–3.301
Thrombus (Yes)	0.190	0.677	1.209	0.495–2.954
Incontinence (Yes)	-1.635	**0.040**	0.195	0.041–0.930
Outpatient Hospitalization				
Comorbidity (No)	1.200	**0.007**	3.319	1.378–7.997
Institution Category (Private)	0.513	**0.011**	1.970	1.176–3.184
Preoperative HDSS	0.046	0.243	1.047	0.969–1.132
Preoperative SHS_HD	0.062	0.061	1.064	0.997–1.136
Disease Grade (Grade 3)	-0.847	**0.023**	2.321	1.123–4.765
Surgical Technique (Non-excisional)	1.116	**0.001**	3.052	1.612–5.775
Gender (Male)	0.273	0.471	1.314	0.625–2.762

(Statistically significant p values were mentioned in bold)

## Discussion

Each surgical modality for the management of Grade III and IV hemorrhoids, including excisional hemorrhoidectomy, stapled hemorrhoidopexy, DG-HAL, and ablative techniques, presents a unique set of advantages and limitations with respect to postoperative outcomes, including pain and recovery time [17–21]. Despite advancements in surgical techniques and perioperative care, significant variability persists in the management of hemorrhoidal disease across different clinical settings. While evidence supports the feasibility of various perioperative practices, their real-world application in clinical setting remains uncertain. A multicenter, nationwide, prospective cohort study was initiated to primarily investigate the impact of different surgical techniques on patient-reported outcome measures in patients with hemorrhoidal disease. This current analysis utilizes data from this ongoing study to evaluate the factors that influenced surgical and perioperative management decisions for patients with Grade III or IV hemorrhoids.

The primary objective of this study was to investigate the factors influencing the choice of surgical technique in patients undergoing hemorrhoidal surgery. Surgical techniques were classified into excisional and non-excisional categories to enable a comprehensive analysis of the factors impacting surgical choice. Univariate analysis revealed that female sex, the presence of comorbidities (ASA ≥ 3), tenesmus, thrombosed hemorrhoids, and higher preoperative HDSS and SHS-HD scores were significantly associated with an increased likelihood of undergoing excisional hemorrhoidectomy. Given that current guidelines recommend surgical excision as the preferred treatment for thrombosed hemorrhoids due to their superior efficacy compared to conservative management, it is not surprising that thrombosis emerged as a significant predictor of the choice of excisional techniques in our analysis [10, 22]. The management of symptomatic hemorrhoids necessitates an individualized approach. Our findings support the established role of conventional hemorrhoidectomy, considered the gold standard for more symptomatic grade 3 and 4 HD [23]. For Grade 4 hemorrhoids, characterized by extensive prolapse and often complicated by thrombosis, excisional hemorrhoidectomy is frequently considered the most effective and reliable procedure by many surgeons. This surgical method not only addresses the physical tissue but also effectively alleviates the significant symptoms associated with Grade 4 hemorrhoids, such as persistent bleeding, prolapse, and pain, which often prove unresponsive to less invasive interventions [24]. Patients with higher HDSS and SHS-HD scores, indicative of more severe disease, were more likely to undergo excisional hemorrhoidectomy in our cohort, aligning with the recognized clinical practice for managing patients with significant symptoms. However, subsequent multivariate analysis revealed that only the presence of thrombosed hemorrhoids among the studied symptoms and signs emerged as a significant predictor of the choice of excisional techniques. This finding aligns with previous studies that suggest thrombosis often necessitates a more aggressive or specific surgical approach due to its potential complications and its impact on clinical outcomes [10–14]. Besides, our findings highlight substantial differences in surgical decision-making practices across various institutions and hospital category was identified as the most significant factor influencing the choice of surgical approach.

This study investigates the factors influencing the selection of perioperative management variables, including anesthesia type, perianal/pudendal analgesia use, and the decision for outpatient discharge. Although our findings suggest an association between smoking status, age, pain and the choices of anesthesia and analgesia techniques, the underlying factors that may drive these clinical decisions are not entirely clear. Further research may be required to explore the determinants of these clinical decisions, including surgeon preference, institutional protocols, and other patient- or institution-related factors. Patients with Grade III hemorrhoids and those with less severe symptoms (lower HDSS and SHS-HD scores) were more likely to undergo surgery under general anesthesia and be discharged on the same day. Furthermore, the utilization of perianal/pudendal analgesia was less frequent in these patient groups. General anesthesia was more frequently employed in the current study, but the choice of anesthesia technique (general vs. spinal/regional) in patients undergoing excisional hemorrhoidectomy might have been influenced by various factors beyond the severity of the complaints, including anesthesiologist preferences or institutional protocols. In addition, the lower preference for outpatient procedures following excisional techniques is not unexpected and may be partly related to concerns about postoperative pain, although current evidence suggests that outpatient management is feasible even after excisional hemorrhoidectomy when adequate multimodal analgesia and patient selection criteria are employed [11–14]. Therefore, the lower rate of outpatient discharge after excisional techniques in our cohort may also reflect institutional preferences and established care pathways rather than an absolute contraindication to same-day discharge.

The study demonstrated a marked heterogeneity in the choice of surgical techniques and perioperative applications across various healthcare institutions. The preference for excisional and non-excisional techniques was significantly influenced by the operating institution. Furthermore, significant inter-institutional variations were observed in the choice of anesthesia type, the use of pudendal or perianal analgesia, and the frequency of outpatient procedures. Although conventional hemorrhoidectomy is widely accepted as the gold standard for severe hemorrhoids, our study demonstrates that the choice of surgical technique is not solely determined by patient characteristics [23]. Significant inter-institutional variations in surgical practices highlight the influence of institutional factors, such as protocols, resources, and surgeon preferences, on surgical decision-making. Despite all centers being tertiary care institutions with presumably similar levels of experience and resource, a significant inter-institutional variation in surgical techniques was observed, which supports the notion that institutional factors are more influential than individual surgeon preferences. While differences in reimbursement policies related to the insurance system in Türkiye between governmental and private institutions may have played a role. In addition, these results may also be questioned because access to advanced technologies and expertise may vary between private and governmental institutions, potentially influencing the observed treatment variations. So, we have further analyzed the hospitals in governmental and private categories separately, which revealed a significant difference between governmental and private hospital categories. Furthermore, inter-institutional variability in surgical technique was also observed within each category, indicating institutional type as the major factor influencing the selection of surgical technique. In addition, as the significant inter-institutional variation observed in the choice of surgical technique, the institution where the surgery was performed significantly influenced the choice of anesthesia, the application of perianal or epidural analgesia, and discharge disposition, which highlights the substantial influence of institutional factors on the overall perioperative management of HD, as well. The observations underlined the substantial influence of institutional factors on the overall care pathway and suggested that institutional-level factors may play an important role in shaping surgical decision-making alongside disease and patient-related factors, although the specific institutional characteristics driving these differences remain to be elucidated in future research. In our opinion, these findings are noteworthy, as they highlight previously undocumented inter-institutional variations in operative and perioperative management of HD. Further research is warranted to investigate the specific factors driving these observed differences.

While the Goligher classification, primarily based on the degree of prolapse of internal hemorrhoids, has been traditionally considered a significant factor in treatment decision-making, our study unexpectedly found that the Goligher classification did not significantly influence the choice of surgical technique [6, 25]. A key limitation of the Goligher classification is its narrow focus on internal hemorrhoid prolapse, which may lead to an overemphasis on surgical intervention and may not adequately reflect the complex pathophysiology and dynamic evolution of HD. Recognizing the limitations of this classification, revisions and novel systems have been proposed considering patient symptomatology and the dynamics of the disease to improve the clinical relevance [15, 26–31]. This study demonstrates a lack of correlation between the Goligher classification and the surgical technique employed, highlighting the limited clinical utility of this classification system in guiding surgical decision-making. However, it should be acknowledged that the lack of observed association between the Goligher grade and surgical technique may also be partially masked by the strong institutional effects identified in this study, as institutional preferences may override classification-guided decision-making. By reflecting real-world clinical practice, current information highlights the limitations of current classification system and emphasizes the need for a new system that prioritizes patient-reported outcomes, correlates treatment outcomes, and guides more effective and personalized management of HD.

A significant limitation of this study is the inherent heterogeneity across institutions. Since the study design did not explicitly focus on inter-institutional variations, the data collected may be insufficient to fully explain the observed differences in surgical techniques. Furthermore, the study design did not clearly address patient selection bias, as it is unclear whether all consecutive patients were included from each participating center.Another limitation of the study design is the lack of comprehensive assessment of factors that may influence surgical decision-making, including surgeon experience, eligibility of surgical devices, patient preferences, and socioeconomic status. These variables may have contributed to the observed variations in surgical technique and other perioperative measures. To fully understand the factors influencing surgical technique selection and to explore the rationale behind surgeons’ decisions regarding perioperative applications, future studies should delve deeper into the decision-making process of individual surgeons such as training, experience, exposure to different techniques, and institutional protocols, patient characteristics, and available resources. This will provide valuable insights into the factors that influence surgical practice and potentially identify opportunities for standardization. Additionally, the exclusion of more than half of the participating centers (11 of 20) due to a minimum case threshold of 10 may have introduced center-level selection bias, potentially limiting the generalizability of our findings to higher-volume institutions. The relatively small number of included institutions (*n* = 9) also limits the power of center-level analyses. The binary logistic regression models used in this study did not account for the hierarchical data structure with patients nested within institutions; multilevel or mixed-effects models would have been more appropriate to disentangle patient-level and institutional-level effects. The absence of surgeon-level variables, such as subspecialty training (coloproctology vs. general surgery), years of experience, and individual technique preferences, represents another important limitation. Furthermore, although hospital category (governmental vs. private) emerged as a significant predictor, the study did not directly measure the specific institutional characteristics underlying this variable, such as reimbursement policies, available technology, staffing structures, or institutional protocols, which limits the interpretability of this finding.

From a clinical perspective, the substantial inter-institutional variability observed in this study suggests that current practice in HD management is not yet fully standardized, even among tertiary care institutions. These findings highlight specific areas where consensus-building efforts may be warranted, including the indications for excisional versus non-excisional techniques, the role of local or regional analgesia as an adjunct to general anesthesia, and the criteria for same-day discharge following hemorrhoidectomy. Future multicenter studies should incorporate multilevel modeling to appropriately account for the hierarchical data structure and should collect detailed surgeon-level and institutional-level variables (e.g., training background, institutional protocols, reimbursement policies, and available technology) to better characterize the sources of practice variation. Collaborative initiatives, such as Delphi-based consensus studies or quality improvement registries, could help translate these observational findings into actionable recommendations for reducing unwarranted practice variation in hemorrhoidal surgery.

As conclusion, despite advancements in surgical techniques and perioperative care, significant inter-institutional variations were observed in the management of grade III and IV HD. Multivariate analysis revealed that while patient and disease-related factors, with the exception of thrombosed HD, did not significantly influence the choice of surgical technique, hospital category emerged as a major determinant of surgical technique and all other aspects of perioperative management, suggesting that institutional factors play a crucial role in shaping clinical practice.

## Supplementary Information

Supplementary Material 1.

## Data Availability

The datasets generated and/or analyzed during the current study are available from the corresponding author on reasonable request.
