# Supplementary material for: Determinants influencing decision-making for operative and perioperative management of grade III and IV hemorrhoidal disease: secondary analysis of a multicenter nationwide prospective cohort study
Source: Langenbecks Arch Surg. 2026 Apr 15;411(1):143. doi: 10.1007/s00423-026-04030-5 (PMC13186793; doi:10.1007/s00423-026-04030-5)
Supplement: Supplementary file 1 — Supplementary Material 1. [file 423_2026_4030_MOESM1_ESM.docx]

STROBE Statement—Checklist of items that should be included in reports of ***cohort studies***

| **Item** | **Recommendation** | **Page** |
| --- | --- | --- |
| **1. Title and Abstract** | Indicate the study’s design with a commonly used term in the title or the abstract: The study title explicitly mentions "Secondary Analysis of a Multicenter Nationwide Prospective Cohort Study." Provide an informative and balanced summary of what was done and what was found: The abstract includes the study objective, design, setting, participants, main outcomes, and key findings. | 1 |
| **2. Background/Rationale** | Explain the scientific background and rationale for the investigation: The introduction provides a comprehensive background on hemorrhoidal disease, its variability in management, and the need for multicenter analyses. | 2 |
| **3. Objectives** | State specific objectives, including any pre-specified hypotheses: Objectives include investigating factors influencing surgical decision-making and perioperative management of Grade III and IV hemorrhoidal disease. | 2 |
| **4. Study Design** | Present key elements of study design early in the paper: Clearly stated as a secondary analysis of a multicenter prospective cohort study. | 3 |
| **5. Setting** | Describe the setting, locations, and relevant dates, including periods of recruitment, exposure, follow-up, and data collection: Conducted between July 2022 and July 2024 at 20 tertiary care or university hospitals across diverse regions in Türkiye. | 3 |
| **6. Participants** | Give the eligibility criteria and sources and methods of selection of participants. Describe methods of follow-up: Inclusion criteria: Patients with Grade III or IV hemorrhoidal disease undergoing surgery. Exclusion criteria include concurrent proctological conditions, history of previous surgeries, or specific comorbidities. | 3 |
| **7. Variables** | Clearly define all outcomes, exposures, predictors, potential confounders, and effect modifiers. Give diagnostic criteria, if applicable: Variables include patient demographics, comorbidities, symptom severity, surgical techniques, anesthesia type, analgesia use, and institutional factors. | 4 |
| **8. Data Sources/Measurement** | For each variable of interest, give sources of data and details of methods of assessment (measurement). Describe comparability of assessment methods if there is more than one group: Data were collected from medical records, patient-reported outcomes (HDSS, SHS-HD), and institutional documentation. | 4 |
| **9. Bias** | Describe any efforts to address potential sources of bias: Efforts include standardizing inclusion criteria and ensuring centers contributed a minimum number of cases to maintain data homogeneity. | 4 |
| **10. Study Size** | Explain how the study size was arrived at: The study included 315 patients, with eligibility determined by pre-defined inclusion and exclusion criteria. | 5 |
| **11. Quantitative Variables** | Explain how quantitative variables were handled in the analyses. If applicable, describe which groupings were chosen and why: Variables were analyzed using logistic regression and stratified by institutional and patient-related factors. | 5 |
| **12. Statistical Methods** | Describe all statistical methods, including those used to control for confounding: Statistical analyses included descriptive statistics, logistic regression, and subgroup analysis. Multicollinearity was assessed using VIF, and significance was set at p < 0.05. | 5 |
| **13. Participants** | Report the number of individuals at each stage of the study (e.g., numbers potentially eligible, examined for eligibility, confirmed eligible, included in the study, completing follow-up, and analyzed): Included 279 patients from 9 institutions after applying exclusion criteria. | 6 |
| **14. Descriptive Data** | Give characteristics of study participants (e.g., demographic, clinical, social) and information on exposures and potential confounders: Participant demographics, comorbidities, symptom severity, and institutional factors are detailed. | 6 |
| **15. Outcome Data** | Report numbers of outcome events or summary measures: Outcome measures include surgical techniques, anesthesia type, and perioperative management decisions, stratified by institution. | 6 |
| **16. Main Results** | Give unadjusted estimates and, if applicable, confounder-adjusted estimates and their precision (e.g., 95% confidence interval). Make clear which confounders were adjusted for and why they were included: Multivariate analyses identified thrombosis and hospital category as significant predictors of surgical decision-making. | 7 |
| **17. Other Analyses** | Report other analyses done—e.g., analyses of subgroups and interactions, and sensitivity analyses: Subgroup analyses were performed based on institutional type (governmental vs. private) and patient-related factors. | 7 |
| **18. Key Results** | Summarize key results with reference to study objectives: Key findings highlight institutional factors as major determinants of surgical decisions, with thrombosed hemorrhoids being the primary patient-related factor. | 8 |
| **19. Limitations** | Discuss limitations of the study, taking into account sources of potential bias or imprecision. Discuss both direction and magnitude of any potential bias: Limitations include heterogeneity across institutions, potential selection bias, and lack of comprehensive assessment of certain variables such as surgeon expertise. | 8 |
| **20. Interpretation** | Give a cautious overall interpretation of results considering objectives, limitations, multiplicity of analyses, results from similar studies, and other relevant evidence: Results emphasize the need for standardization in hemorrhoidal disease management and further research into institutional influences. | 9 |
| **21. Generalizability** | Discuss the generalizability (external validity) of the study results: Findings are generalizable to tertiary care settings but may not apply to primary care or non-tertiary institutions. | 9 |
| **22. Funding** | Give the source of funding and the role of the funders for the present study and, if applicable, for the original study on which the present article is based: Not explicitly mentioned; authors declare no financial or non-financial competing interests. | 10 |
| **23. Ethical Approval** | Indicate whether ethical approval was obtained, and outline the consent process: Ethical approval granted by Istanbul Medipol University Research Committee (Protocol number: E-10840098-772.02-3634). All participants provided informed consent. | 10 |

*Give information separately for exposed and unexposed groups.
